# Predicting the impact of insecticide-treated bed nets on malaria transmission: the devil is in the detail

**DOI:** 10.1186/1475-2875-8-256

**Published:** 2009-11-16

**Authors:** Weidong Gu, Robert J Novak

**Affiliations:** 1 Division of Infectious Diseases, University of Alabama, Birmingham, Alabama 35294, USA

## Abstract

**Background:**

Insecticide-treated bed nets (ITNs), including long-lasting insecticidal nets (LLINs), play a primary role in global campaigns to roll back malaria in tropical Africa. Effectiveness of treated nets depends on direct impacts on individual mosquitoes including killing and excite-repellency, which vary considerably among vector species due to variations in host-seeking behaviours. While monitoring and evaluation programmes of ITNs have focuses on morbidity and all-cause mortality in humans, local entomological context receives little attention. Without knowing the dynamics of local vector species and their responses to treated nets, it is difficult to predict clinical outcomes when ITN applications are scaled up across African continent. Sound model frameworks incorporating intricate interactions between mosquitoes and treated nets are needed to develop the predictive capacity for scale-up applications of ITNs.

**Methods:**

An established agent-based model was extended to incorporate the direct outcomes, e.g. killing and avoidance, of individual mosquitoes exposing to ITNs in a hypothetical village setting with 50 houses and 90 aquatic habitats. Individual mosquitoes were tracked throughout the life cycle across the landscape. Four levels of coverage, i.e. 40, 60, 80 and 100%, were applied at the household level with treated houses having only one bed net. By using Latin hypercube sampling scheme, parameters governing killing, diverting and personal protection of net users were evaluated for their relative roles in containing mosquito populations, entomological inoculation rates (EIRs) and malaria incidence.

**Results:**

There were substantial gaps in coverage between households and individual persons, and 100% household coverage resulted in circa 50% coverage of the population. The results show that applications of ITNs could give rise to varying impacts on population-level metrics depending on values of parameters governing interactions of mosquitoes and treated nets at the individual level. The most significant factor in determining effectiveness was killing capability of treated nets. Strong excito-repellent effect of impregnated nets might lead to higher risk exposure to non-bed net users.

**Conclusion:**

With variabilities of vector mosquitoes in host-seeking behaviours and the responses to treated nets, it is anticipated that scale-up applications of INTs might produce varying degrees of success dependent on local entomological and epidemiological contexts. This study highlights that increased ITN coverage led to significant reduction in risk exposure and malaria incidence only when treated nets yielded high killing effects. It is necessary to test efficacy of treated nets on local dominant vector mosquitoes, at least in laboratory, for monitoring and evaluation of ITN programmes.

## Background

Malaria campaigns in tropical Africa have been refuelled due to increasingly available funds from multiple international agencies. Insecticide-treated nets (ITNs) and indoor residual spray (IRS) are the major tactics for combating malaria mediated by three major malaria vectors, *Anopheles gambiae*, *Anopheles arabiensis *and *Anopheles funestus*, in sub-Saharan Africa. Scale-up applications of ITNs in particular are highlighted because it not only protects users, but also non-users through insecticidal and/or repellent effects concurred by treated nets. The latter requires a high coverage so that the whole community can benefit[1].

ITNs influence malaria transmission by killing and/or diverting mosquitoes away from the net user and house with treated nets[2]. In tropical Africa, different vector species vary substantially in host-seeking behaviours, and consequently respond differently to use of ITNs[2,3]. Additionally, studies have shown that extensive use of ITNs could result in reduced susceptibility of *An. gambiae *to treated nets [4]. Therefore, it is important to monitor changes in mosquito populations for evaluation of application of treated nets in the field. Studies of experimental huts revealed varying effects of ITNs due to variabilities in host-seeking behaviours and use of different insecticides [5-9]. For example, pyrethroid-treated nets induced 40% mortality on *An. arabiensis *in West Africa [10], compared to estimated 90% mortality reported in Cameroon[5]. For the long-lasting insecticidal nets, 74 and 63% mortality against *An. funestus *and *An. gambiae*, respectively, were observed in Tanzania [7]. These variations of ITN impacts on mosquitoes imply that results of randomized community trials might be circumstantial with limited potentials for generalization. Under these circumstances, it is concerned that the emphasis on increased ITN coverage for combating malaria in Africa might fail to generate anticipated results in African countries, where entomological and epidemiological conditions vary tremendously[11].

Much monitoring and evaluation programmes have focused on metrics of coverage, morbidity and all-cause mortality in humans by randomized community trials while entomological surveillance mentioned above is largely lacking[12]. To predict the impact of ITNs on malaria transmission, it is essential to use models which represent processes of mosquito's reactions to treated nets[13]. Previous models assumed a uniform contact structure between mosquitoes and hosts across the landscape, i.e. hosts are equally available to any mosquitoes no matter where their locations are[14,15]. Despite mathematically convenient, this assumption needs to be revisited because empirical data indicate limited ranges of flight and perception [16-18]. Clearly, mosquitoes more likely bite on available hosts nearby rather than distanced ones. From the perspective of individual mosquitoes, the probability of locating a host is a function of the number of available hosts in the proximity, which in turn is defined on the range of mosquito flight and perception[19]. To represent local search process, spatially explicit frameworks are needed to track distances between individual host-seeking mosquitoes and humans across the landscape. By formulating a notion of the localized availability, an agent-based model was developed to obtain insights regarding the impact of source reduction on malaria transmission [20]. In this paper, the existent model was extended to incorporate plausible responses of individual mosquitoes to treated nets for evaluation of ITNs on malaria transmission. The relative role of parameters governing responses to ITNs at individual mosquito level was assessed. Although this study had a focus on *An. gambiae *complex in Africa, sensitivity analysis of parameter combinations encompassed a wide range of scenarios which might fit specific human-mosquito interactions in the presence of ITNs encountered in different malaria settings. The implication of this modelling exercise highlights the necessity of entomological surveillance for evaluation of scale-up applications of ITNs.

## Methods

**Figure 1:**
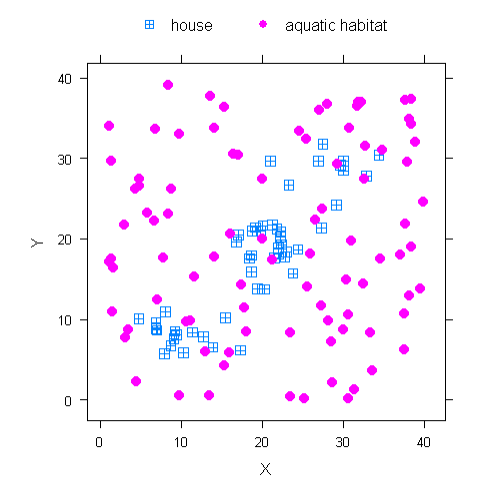
**Schematic of the hypothetical village with 50 houses and 90 habitats**.

A hypothetical village was created in a grid-based landscape with 40 × 40 grids (50 × 50 m each). Fifty houses were clumped along the left-bottom to right-top diagonal and 90 aquatic habitats were randomly distributed across the landscape (Figure 1). Here, alternative hosts for blood feeding, e.g. cattles which were studied by others[14,21], were not included, and the only blood source was humans in the houses. All the houses and aquatic habitats were assumed to be identical in their attractiveness to mosquitoes. The number of residents in each house was a random number with an average of six and a minimum of two, i.e. the father and mother.

The dynamics of mosquito populations was detailed previously [22]. Here a brief description is provided. Eggs, larvae and pupae were simulated at individual habitats with daily transitional rates of 0.3, 0.2 and 0.3, respectively. The daily survival rate of immature was assumed as 0.8[23]. By setting the carry capacity of aquatic habitats as 5,000 eggs, it was meant that ovipositing females would avoid laying eggs in the saturated habitats. Emergent female mosquitoes were individually tracked. Based on their developmental state and blood feeding, females were classed into one of three categories: newly emerged, host-seeking and gravid mosquitoes. To simulate female mosquitoes in their search for hosts/oviposition sites, the same rules were adopted (in terms of ranges of flight and perception) for host-seeking and ovipositing mosquitoes. The perception area was defined as eight adjacent grids of the focal mosquitoes, i.e. hosts/habitats in this area were immediately available. Otherwise, mosquitoes would engage a random flight by moving randomly into one of the adjacent grids, and search flight did not stop until the maximal flight length was reached or the resource was located. This foraging scheme was consistent with a two-phase flight search patterns characterized as appetential and consummatory flight [24]. The advantages of this scheme are its readiness to represent ranges of perception and flight separately by sizes of grids and the number of grids in the flight path, and these parameters can be empirically measured in the field [25,26]. The grid size was set as 50 m, equivalent to the perception range of 2.25 (150 × 150 m^2^) ha to reflect limited perceptual ranges of *An. gambiae *observed in the field[17]. Two virtual mosquitoes were constructed with the maximal flight lengths of 1 and 3 grids, respectively. Malaria transmission was simulated as a susceptible-infected SI model. Each infection was presumed to last for 100 days, equivalent to the recovery rate 0.01, and restored to be susceptible[27]. Transmission between mosquitoes and hosts was probabilistic: once bitten by an infectious mosquito a susceptible person became infected at the rate 0.5, and uninfected mosquitoes became infected when biting infected persons at the rate 0.15. The incubation in infected persons and extrinsic incubation period in infected mosquitoes were assumed as 15 and 10 days, respectively[28,29].

### Coverage of ITNs

Four levels of household coverage of 40, 60, 80 and 100% were evaluated. In each case, only one ITN bed net was assigned to the randomly selected houses, and two randomly selected persons in the house were protected by the net. Note that coverage here was in line with the outcome indicator of ITNs adopted Roll Back Malaria Partnership as the proportion of households with at least one treated net [30], in contrast to the coverage defined as the proportion of populations sleeping under treated nets[14]. A survey in South Africa showed that the pregnancy rate was 0.08 among mothers and children under five accounted for 30% of the population [31]. Therefore, the policy of protection of only the vulnerable pregnant women and children under five would be equivalent to 60% coverage of households in this setting.

### Modelling responses of individual mosquitoes to ITNs

The outcomes of host-seeking mosquitoes entering houses with a treated net can be categorized into one of three mutually exclusive categories: 1) killed; 2) deterred by excito-repellence; and 3) succeeded in feeding[14]. These outcomes were represented in a sequence of processes characterized as following. First, a proportion *α *of host-seeking mosquitoes locating the treated house were diverted and might continue to seek blood from the nearest house based on a parameter *h *describing how many houses the diverted mosquito could search. Second, for the mosquitoes entering the treated house, the mortality concurred by ITN was μ. Third, the survival mosquitoes fed randomly on residents with the probability of β succeeding in feeding on net users. If feeding was unsuccessful on the net user, the mosquitoes would switch to another resident. Feeding on non-users was presumably always successful. Therefore, β was a measure of personal protection of net users from being bitten. By varying values of these parameters, a wide range of scenarios entailing various entomological and epidemiological settings encountered in the field could be evaluated. For example, use of untreated nets would correspond to scenarios of no repellency and mortality while some degrees of protection against feeding, i.e. *α *= 0, μ = 0 and β >0.

For houses without treated nets, mosquitoes would enter the houses and feed randomly on residents. There was no assumption of density-dependent regulation of blood feeding in our model. Mosquitoes could continue to move up to three grids in a day when hosts or aquatic habitats were not in the neighbouring grids. Note this flight movement did not include movement of diverted mosquitoes which automatically moved to the nearest house no matter whether it was located in the same grid or not. Because of clumped distribution of houses, diversion dispersal was most likely within the same grid. Unlike others[14], there were no mortality and/or diversion in the absence of treated nets in this study. However, addition of these baseline effects, albeit affecting absolute numbers of predicted mosquito populations, had little effects on our comparisons of intervention scenarios consisting of a spectrum of parameter values and ITN coverage. The framework of agent-based models provides a great flexibility for describing intricacies and details of individual mosquitoes. For example, subsequent movements of diverted mosquitoes by ITNs can be readily tracked.

### Statistical analyses

Given the variability and uncertainty about estimation of mosquito responses to ITNs, vigorous sensitivity analysis was conducted to examine the relative influences of parameters. Specifically, four parameters *μ*, *α, β *and *h *were sampled from the ranges of 0~0.9, 0.2~0.9, 0.3~1.0 and 0~5, respectively. Latin hypercube sampling (R system[32], lhs package, http://cran.r-project.org/web/packages/lhs/lhs.pdf) was conducted to obtain representative samples from the hyper-parameters domain consisting of *μ, α, β *and *h*. Specifically, 50 sets of representative parameters were generated by choosing from uniform distributions bounded by the minimum and maximum values of these parameters. Each parameter set was used to run the simulation once. Averages of mosquito abundances and malaria incidence of net users and non-net users over the period of the intervention (from day 150 to 300) were recorded. For assessing the relative influence of parameters on malaria transmission, regression tree was applied. Regression tree models explain a continuous dependent variable by categorical and numeric independent variables. Regression tree is a powerful alternative to multiple regression models with better elucidation of patterns in the data and easier to interpret due to its allowance of higher order interactions in the parametric analysis. Unlike conventional statistical analyses, which assume linearity and additivity, a regression tree is inherently non-parametric and can handle highly skewed and multi-modal data[33]. The fundamental idea of regression tree is to recursively split the data according to dependent variable into mutually exclusive and homogeneous as possible groups by repeatedly using independent variables. To keep the tree reasonably small, pruning is applied to reduce the fully-grown tree to the desired size. Each group is typically characterized by mean value of dependent variable. Trees are represented graphically with the root node representing the original data and the leaves the final groups. Statistical analyses and multi-panel figures were carried out in the R system [32]. In particular RPART package [34]was used for regression tree model. To determine optimally sized tree, 10-fold cross-validations were run on the data which entailed 10 random divisions of the data into 90% learning and 10% test sets. The threshold of complexity parameter value was 0.01.

### Simulations

The model was formulated using object-oriented programming C++ and was run on a Dell dual-core CPU Xeon 5100 computer. Simulations were carried out on each scenario of parameter combinations with an initial population of 20,000 gravid mosquitoes originating from randomly selected houses. Fifty percent of the residents were assumed to be infected with varying dates of infection of -100 to -1 day. Abundances of mosquito populations reached plateaus after 100 days and treatments of ITNs were introduced at 150 days. Each simulation lasted for 300 days. Averages of total mosquito abundance, daily EIR and the incidence and prevalence of malaria were recorded. Daily EIRs were derived by summarizing all host-seeking mosquitoes with infectious status on a particular day. Malaria incidence in humans was summarized as the number of new infections over the period from day 150 to 300. Impacts of ITNs (*I*) on mosquito densities, EIR and malaria incidence were calculated as *I *= 100 (m_0_-m)/m_0_, where m and m_0 _were the corresponding measures under ITN treatments and no bed nets, respectively.

## Results

There was a noticeable gap in ITN coverage between household and individual levels (Table 1). The average of household residents was 3.7 (sd = 1.2) with 56% of households having more than three persons. The policy of only one bed net per household and two persons sleep under as considered here only achieved a protection level of 55% population for the complete coverage of households.

**Table 1:** Settings of ITN coverage at the person and household level

Household coverage (%)	Personal coverage (%)	Number of net users	Number of non users
0	0	0	185
40	23	42	143
60	34	62	123
80	44	82	103
100	55	102	85

**Figure 2:**
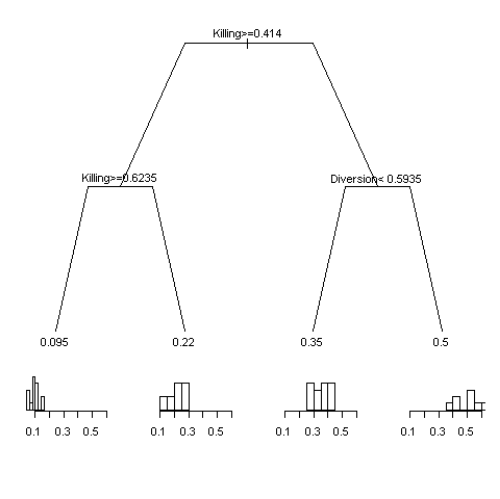
**Regression tree of malaria incidence as a function of killing, diversion and protection effects of insecticide-treated bed nets under the coverage of 80% households**. For each split, the branch on the left meets the criterion (text on the split). At the end of splits (called leaves) were averages of malaria incidence of the partitioned data sets along with corresponding histograms.

Variations in mosquito's responses to ITNs gave rise to a substantial degree of variation in malaria incidence ranging from 0.1 to 0.5 under the ITN coverage of 80% (Figure 2). Regression tree reveals intricate influences of insecticidal and repellent effects of ITNs on malaria incidence. Unsurprisingly, insecticidal effect was the most influencing factor, followed by diversion. If killing was greater than 0.6, for example, protection of net users by ITNs in 80% coverage had a limited effect, e.g. incidence reduced from 0.21 to 0.16 when protection were larger than 0.68. Interestingly, the coefficient of protection effect of net users did not produce noticeable effect on malaria incidence of the whole population. However, a statistical test revealed that increased levels of personal protection of ITNs could reduced malaria incidence only in net users (F = 22.9, df = 1 and 198, p < 0.01). Numbers of houses *h *visited by diverted mosquitoes appear to have no significant effect.

**Figure 3:**
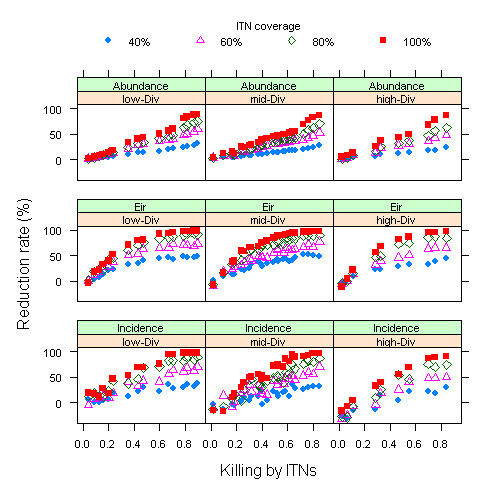
**Reduction rates (%) of mosquito abundances (upper panels), EIR (mid panels) and Incidence as a function of insecticidal effect conditional on three levels of diversion effect (low 0~0.33, medium 0.33~0.66 and high 0.66~1)**.

It is notable that mosquito abundances responded differently from EIR and malaria incidence related to insecticidal effect of INTs (Figure 3). Surprisingly, impacts on EIR and incidence were more sensitive to variability in the insecticidal effect than mosquito abundances, especially at the low range of diversion effect. The cause of this deviation was attributed to the fact that EIR and incidence directly measures contact rates between mosquito and humans, while abundance measured the overall mosquito population. ITNs target the contact rate by providing protection to net users and non-users alike and, therefore, have more significant effects on EIR and incidence than mosquito abundances. Additionally, reduction rates of mosquito abundance were lower with increased levels of diversion, especially at the low spectrum of insecticidal effect. For highly repellent and limited killing nets, application of INTs increased malaria incidence (reduction rates could be < 0, the bottom-right panel).

**Figure 4:**
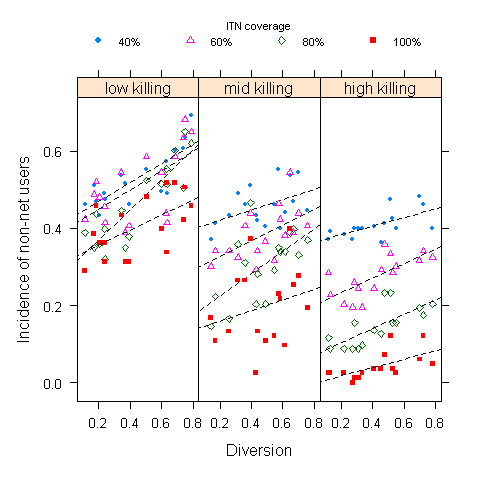
**Incidence of malaria in unprotected population as a function of diversion effect of ITNs conditional on 3 levels of insecticidal effect (low 0~0.3, mid 0.3~0.6, high 0.6~0.9)**.

The communal protection concurred by INTs to non-bed net users was influenced by repellent and killing effects (Figure 4). Unsurprisingly, increased levels of repellency enhanced the risk of infection to non-users. Only with strong insecticidal effects to suppress mosquito populations, malaria incidence declined in non-users.

## Discussion

This model provides a mechanism to link outcomes of ITNS on individual mosquitoes to population-level risk exposure and malaria incidence. A wide range of impacts on population-level malaria risk exposure and incidence were observed corresponding to variations in the responses of individual mosquitoes. Increased coverage of treated nets led to reduced disease burden only when ITNs resulted in significant mortality rates. The insecticidal effect played the primary role in effectiveness of ITN programmes while repellency and protection were secondarily important. In sub-Saharan Africa, where *An. gambiae*, *An. arabiensis *and *An. funestus *vary considerably in host-seeking habits, e.g. timing and sites of feeding, studies of experimental huts provide direct measures of repellency, insecticidal and protection against feeding of treated nets. Various impacts of ITNs were reported for different vector species from experimental hut evaluations in Africa [5,7,10]. Resistance to pyrethroids can significantly alleviate mosquito mortality and protection against feeding in Benin[9]. Therefore, variations in biological traits, e.g. biting habit, of vector species in Africa make it hard to predict applications of ITNs in large areas.

There is a big gap in coverage levels based on households and individuals. The complete coverage of households with one nets reached modest levels c. 50% personal coverage. Multiple Indicator Cluster Surveys (MICS) and Demographic and Health Surveys (DHS) in Africa show that the average size of households is c. five persons[35]. Therefore, a large number of treated nets are needed to reach desirable coverage at the individual level. Simulation results show that increased coverage of ITNs indeed generated noticeable reductions in mosquito abundance, EIR and malaria incidence, especially when the insecticidal effect of ITNs was high (Figure 3), consistent with other reports based on mathematical models[14,21]. The importance of scale-up coverage of ITNs beyond vulnerable population (children under five years of age and pregnant women) has been emphasized as the priority for combating malaria in tropical Africa[36].

The community-wide impact of INTs lies in the protection to non-users due to substantial killing of mosquitoes. However, this effect is hinged upon an intricate balance between killing and excite-repellent effect of ITNs. Other things being equal, increased levels of repellency of ITNs enhanced the risk of non-users (Figure 4). ITNs with strong deterrence and less killing resulted in diversion of more host-seeking mosquitoes to residents unprotected by ITNs.

Agent-based models provide a powerful tool to incorporate entomological details relevant to malaria transmission. For example, exposure to pyrethroid insecticide-impregnated nets might not suffice to kill mosquitoes, but lower the life span as evidenced by ovarian age-grading technique in Tanzania[6]. This elevated mortality as a function of a number of exposure can readily be represented in agent-based models. A wide spectrum of scenarios of combined effects of ITNs on individual mosquitoes can be examined which may arise due to variabilities in vector species and the manner of treated nets being used. For example, the protection of untreated nets can be examined by assigning levels of protection against feeding based on field observations while assuming null insecticidal and repellent effects. By measuring corresponding parameters from well-designed experimental studies, it provides mechanistic understandings regarding observed variations in effectiveness of ITNs in different situations.

The important implication of this study is that increased coverage of ITNs might not obtain expectations and goals in scale-up applications of large areas due to variations in local entomological context, including vector species and their responses to treated nets. It is necessary to point out that knowledge of host-seeking behaviours and responses of mosquitoes to treated nets is far from complete for generalization of limited numbers of community trials. Monitoring and evaluation of incidence and/or all cause child mortality through randomized community trials provide the most strong incentive for motivation of international resources. Efficacy tests, at least in laboratory, of locally dominant mosquitoes to treated nets following the standard protocols [37] are necessary for scale-up applications.

## Conclusion and recommendations

Global efforts for combating and eliminating malaria in sub-Saharan Africa have set the focus on increase of ITN coverage, from 60% pregnant women and children under five years of age, of the Abuja Declaration and Plan Action [38] to the full coverage of populations at risk [30]. By taking account of the complicated entomological context, this study indicates that increased coverage did not necessarily translate to reduced risk exposure and disease burden due to variabilities in host-seeking behaviours of major vector mosquitoes. Moreover, efficacy of treated nets varies with insecticides, duration of use and human behaviours. From the biological viewpoint, this modelling exercise highlights the complexity of scale-up applications of ITNs for malaria management and elimination in sub-Saharan Africa.

## Conflicts of interests

The authors declare that they have no competing interests.

## Authors' contributions

WG designed the study protocol and carried out the study and data analysis; WG and RJN interpreted the results; both authors read and approved the final manuscript. WG is guarantor of the paper.

## Ethical approval

Not required.

## References

[B1] Curtis CF Jana-Kara B Maxwell CA Insecticide treated nets: impact on vector populations and relevance of initial intensity of transmission and pyrethroid resistance J Vector Borne Dis 2003 40 1 8 15119065

[B2] Takken W Do insecticide-treated bednets have an effect on malaria vectors? Trop Med Int Health 2002 7 1022 1030 10.1046/j.1365-3156.2002.00983.x 12460393

[B3] Lindblade KA Gimnig JE Kamau L Hawley WA Odhiambo F Olang G Ter Kuile FO Vulule JM Slutsker L Impact of sustained use of insecticide-treated bednets on malaria vector species distribution and culicine mosquitoes J Med Entomol 2006 43 428 432 10.1603/0022-2585(2006)043[0428:IOSUOI]2.0.CO;2 16619629

[B4] John R Ephraim T Andrew A Reduced susceptibility to pyrethroid insecticide treated nets by the malaria vector *Anopheles gambiae *s.l. in western Uganda Malar J 2008 7 92 10.1186/1475-2875-7-92 18503715 PMC2432068

[B5] Chouaibou M Simard F Chandre F Etang J Darriet F Hougard JM Efficacy of bifenthrin-impregnated bed nets against *Anopheles funestus *and pyrethroid-resistant *Anopheles gambiae *in North Cameroon Malar J 2006 5 77 10.1186/1475-2875-5-77 16961938 PMC1584243

[B6] Magesa SM Wilkes TJ Mnzava AE Njunwa KJ Myamba J Kivuyo MD Hill N Lines JD Curtis CF Trial of pyrethroid impregnated bednets in an area of Tanzania holoendemic for malaria. Part 2. Effects on the malaria vector population Acta Trop 1991 49 97 108 10.1016/0001-706X(91)90057-Q 1680284

[B7] Malima RC Magesa SM Tungu PK Mwingira V Magogo FS Sudi W Mosha FW Curtis CF Maxwell C Rowland M An experimental hut evaluation of Olyset nets against anopheline mosquitoes after seven years use in Tanzanian villages Malar J 2008 7 38 10.1186/1475-2875-7-38 18307802 PMC2267806

[B8] Mathenge EM Gimnig JE Kolczak M Ombok M Irungu LW Hawley WA Effect of permethrin-impregnated nets on exiting behavior, blood feeding success, and time of feeding of malaria mosquitoes (Diptera: Culicidae) in western Kenya J Med Entomol 2001 38 531 536 10.1603/0022-2585-38.4.531 11476333

[B9] N'Guessan R Corbel V Akogbeto M Rowland M Reduced efficacy of insecticide-treated nets and indoor residual spraying for malaria control in pyrethroid resistance area, Benin Emerg Infect Dis 2007 13 199 206 10.3201/eid1302.060631 17479880 PMC2725864

[B10] Oxborough RM Mosha FW Matowo J Mndeme R Feston E Hemingway J Rowland M Mosquitoes and bednets: testing the spatial positioning of insecticide on nets and the rationale behind combination insecticide treatments Ann Trop Med Parasitol 2008 102 717 727 10.1179/136485908X337553 19000389

[B11] Smith DL Hay SI Noor AM Snow RW Predicting changing malaria risk after expanded insecticide-treated net coverage in Africa Trends Parasitol 2009 25 511 516 10.1016/j.pt.2009.08.002 19744887 PMC2768685

[B12] Lengeler C Insecticide-treated bed nets and curtains for preventing malaria Cochrane Database Syst Rev 2004 CD000363 15106149 10.1002/14651858.CD000363.pub2

[B13] Killeen GF Kihonda J Lyimo E Oketch FR Kotas ME Mathenge E Schellenberg JA Lengeler C Smith TA Drakeley CJ Quantifying behavioural interactions between humans and mosquitoes: evaluating the protective efficacy of insecticidal nets against malaria transmission in rural Tanzania BMC Infect Dis 2006 6 161 10.1186/1471-2334-6-161 17096840 PMC1657018

[B14] Killeen GF Smith TA Ferguson HM Mshinda H Abdulla S Lengeler C Kachur SP Preventing childhood malaria in Africa by protecting adults from mosquitoes with insecticide-treated nets PLoS Med 2007 4 e229 10.1371/journal.pmed.0040229 17608562 PMC1904465

[B15] Le Menach A Takala S McKenzie FE Perisse A Harris A Flahault A Smith DL An elaborated feeding cycle model for reductions in vectorial capacity of night-biting mosquitoes by insecticide-treated nets Malar J 2007 6 10 10.1186/1475-2875-6-10 17254339 PMC1794417

[B16] Gillies MT Wilkes TJ The range of attraction of single baits fro some West African mosquitoes Bull Entomol Res 1970 60 225 235 10.1017/S000748530004075X 22894841

[B17] Gillies MT Wilkes TJ The range of attraction of animal baits and carbon dioxide for mosquites Bull Entomol Res 1972 61 389 404 10.1017/S0007485300047295 4393126

[B18] Gillies MT Wilkes TJ The range of attraction of birds as baits for some West African msoquitoes Bull Entomol Res 1974 63 573 581 10.1017/S0007485300047817 22894841

[B19] Gu W Regens JL Beier JC Novak RJ Source reduction of mosquito larval habitats has unexpected consequences on malaria transmission Proc Natl Acad Sci USA 2006 103 17560 17563 10.1073/pnas.0608452103 17085587 PMC1634411

[B20] Gu W Novak RJ Agent-based modelling of mosquito foraging behaviour for malaria control Trans R Soc Trop Med Hyg 2009 103 11 1105 12 10.1016/j.trstmh.2009.01.006 19200566 PMC2818421

[B21] Killeen GF Smith TA Exploring the contributions of bed nets, cattle, insecticides and excitorepellency to malaria control: a deterministic model of mosquito host-seeking behaviour and mortality Trans R Soc Trop Med Hyg 2007 101 867 880 10.1016/j.trstmh.2007.04.022 17631372 PMC1949412

[B22] Gu W Novak RJ Agent-based modeling of mosquito foraging for malaria control Trans R Soc Trop Med Hyg 2009 103 1105 1112 10.1016/j.trstmh.2009.01.006 19200566 PMC2818421

[B23] Edillo FE Toure YT Lanzaro GC Dolo G Taylor CE Survivorship and distribution of immature Anopheles gambiae s.l. (Diptera: Culicidae) in Banambani village, Mali J Med Entomol 2004 41 333 339 10.1603/0022-2585-41.3.333 15185933

[B24] Bidlingmayer WL The measurement of adult mosquito population changes--some considerations J Am Mosq Control Assoc 1985 1 328 348 2906674

[B25] Bidlingmayer WL Hem DG The range of visual attraction and the effect of competitive visual attractants upon mosquito flight Bull Entomol Res 1980 70 321 342 10.1017/S0007485300007604

[B26] Gillies MT Studies on the dispersion and survival of *Anopheles gambiae *Giles in East Africa, by means of marking and release experiments Bull Entomol Res 1961 52 99 127 10.1017/S0007485300055309

[B27] Smith DL Dushoff J McKenzie FE The risk of a mosquito-borne infection in a heterogeneous environment PLoS Biol 2004 2 e368 10.1371/journal.pbio.0020368 15510228 PMC524252

[B28] Gu W Mbogo CM Githure JI Regens JL Killeen GF Swalm CM Yan G Beier JC Low recovery rates stabilize malaria endemicity in areas of low transmission in coastal Kenya Acta Trop 2003 86 71 81 10.1016/S0001-706X(03)00020-2 12711106

[B29] Lines JD Wilkes TJ Lyimo EO Human malaria infectiousness measured by age-specific sporozoite rates in *Anopheles gambiae *in Tanzania Parasitology 1991 102 Pt 2 167 177 10.1017/S0031182000062454 1852484

[B30] RBM P Guidelines for core population-based indicators Technical Paper - RBM/WG/2009/TP:2001 2009

[B31] Madhavan S Schatz EJ Coping with change: Household structure and composition in rural South Africa, 1992 - 2003 Scandinavian Journal of Public Health 2007 35 85 93 10.1080/14034950701355627 PMC2830111 17676508

[B32] Team RDC R: A language and environment for statistical computing 2008 R Foundation for Statistical Computing. Vienna, Austria

[B33] Breiman L Friedman J Stone CJ Olshen RA Classification and Regression Trees 1984 London, UK: Taylor & Francis, Inc

[B34] Therneau TM Atkinson EJ An introduction to recursive partitioning using the RPART routines Technical Report 61 1997 Rochester, Minnesota: Mayo Clinic, Section of Biostatistics

[B35] Miller JM Korenromp EL Nahlen BL R WS Estimating the number of insecticide-treated nets required by African households to reach continent-wide malaria coverage targets JAMA 2007 297 2241 2250 10.1001/jama.297.20.2241 17519414

[B36] WHO Insecticide-treated mosquito nets: a WHO position statement 2007 programme GM. WHO Geneva

[B37] WHO Guidelines for laboratory and field testing of long-lasting insecticidal mosquito nets 2005 Geneva: World Health Organization

[B38] Yamey G Roll Back Malaria: a failing global health campaign BMJ 2004 328 1086 1087 10.1136/bmj.328.7448.1086 15130956 PMC406307

